# Supplementary material for: Mapping of histone-binding sites in histone replacement-completed spermatozoa
Source: Nat Commun. 2018 Sep 24;9:3885. doi: 10.1038/s41467-018-06243-9 (PMC6155156; doi:10.1038/s41467-018-06243-9)
Supplement: Supplementary file 1 — Supplementary Infomation [file 41467_2018_6243_MOESM1_ESM.pdf]

Supplementary information for

Mapping of histone-binding sites in histone replacement-completed spermatozoa

Keisuke Yoshida, Masafumi Muratani, Hiromitsu Araki, Fumihito Miura, Takehiko Suzuki, Naoshi Dohmae, Yuki Katou, Katsuhiko Shirahige, Takashi Ito, and Shunsuke Ishii

Supplementary Figures

Supplementary Fig. 1 | The principle and usability of SCSA.

Supplementary Fig. 2 | Application of SCSA to analysis of mouse sperm cells.

Supplementary Fig. 3 | Measurement of quantitative ratio of histone H3 between total sperm and HRCS fractions by LC-MS assay.

Supplementary Fig. 4 | Raw data of H3 ChIP-seq results using total sperm and HRCS in representative genomic regions.

Supplementary Fig. 5 | Analysis for genomic feature of H3-binding site distributions.

Supplementary Fig. 6 | Correlation of H3-binding sites and DNA methylation status.

Supplementary Fig. 7 | Binding motif and epigenetic signature for H3 target genes in total sperm and HRCS.

Supplementary Fig. 8 | Relationship between H3 target genes in sperm and epigenetic modification in 4-cell stage.

Supplementary Fig. 9 | Dynamics of epigenetic mark in H3 target genes during early embryogenesis.

Supplementary Fig. 10 | Dynamics of expression profile in H3 target genes during early embryogenesis.

Supplementary Fig. 11 | Analysis for the presence of H3K9me2/3 marks for category_H genes in HRCS.

Supplementary Fig. 12 | Uncropped data of western blotting and agarose gel electrophoresis.

Supplementary Table 1 | List of primers used in this study

Supplementary Table 2 | Query codes of published datasets used in this study

a

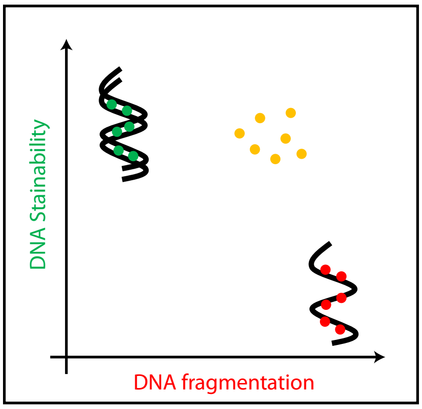

SCSA (sperm chromatin structure assay) is method of staining sperm by DNA dye, Acridine Orange (AO).

AO +dsDNA >> Green

AO +ssDNA >> Red

Now, SCSA is generally used for diagnostic assessment of human sperm quality.

b

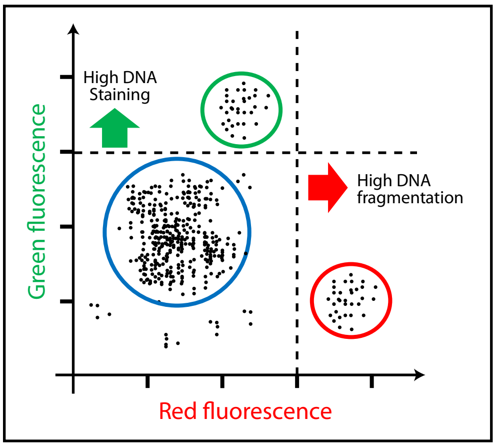

1. Normal sperm fraction
> Normal DNA condensation and no DNA damage

2. HDS (High DNA Stainability)
> Sperm nuclears have Lower DNA condensation
> HDS is recognized as "immaure sperm" in human SCSA.

AO preferentially binds to histone- remaining region rather than protamine- associated DNA.

3. DFI (DNA fragmentation index)
> Sperm nuclears have DNA damege.

HDS(%) and DFI(%) are negatively correlated with pregnancy success rate in human.

Supplementary Fig. 1 | The principle and usability of SCSA. a, The principle of SCSA. **b,** The schematic view of SCSA result (not actual result). These figures were prepared by reference to Supplementary Ref. 1.

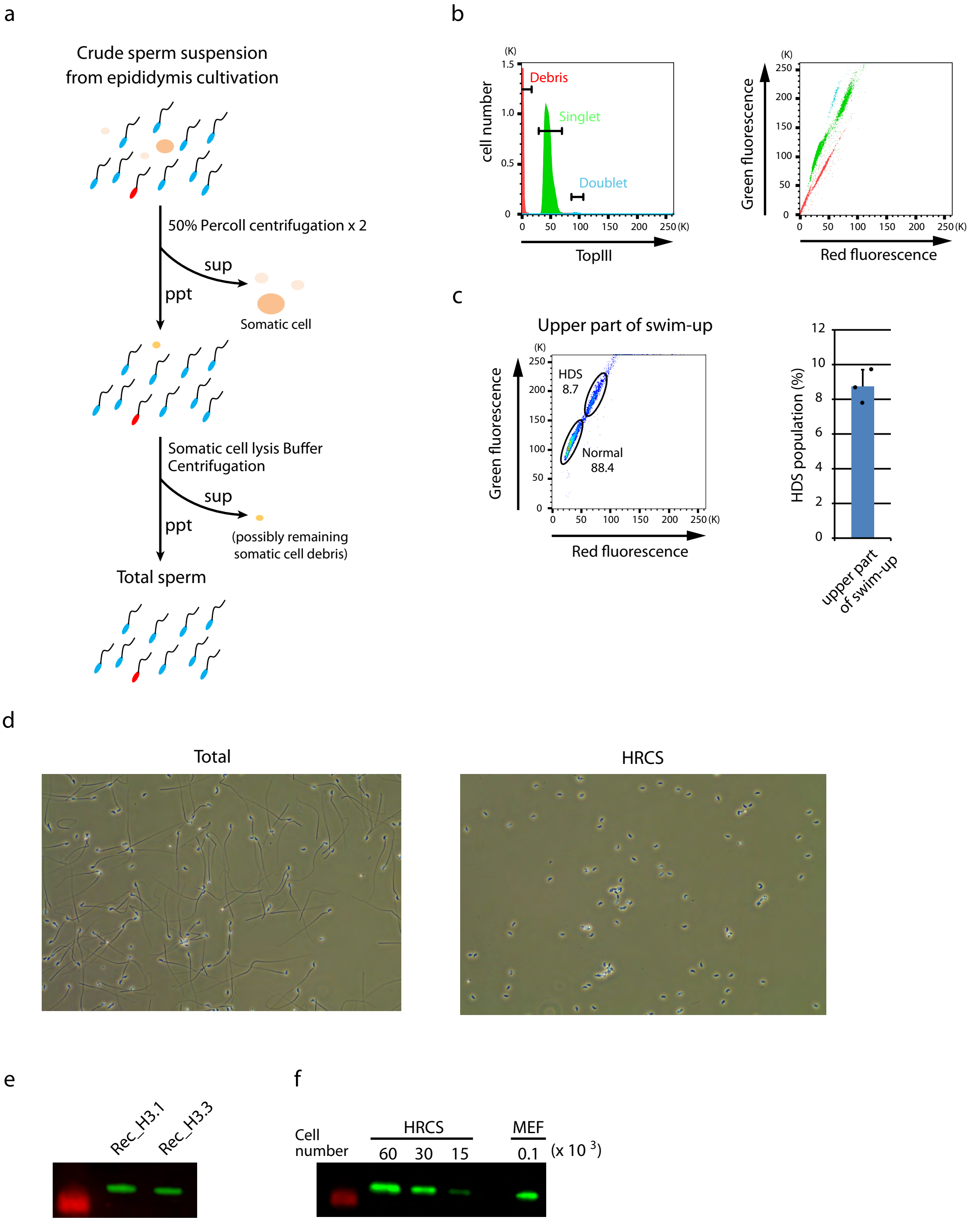

Supplementary Fig. 2 | Application of SCSA to analysis of mouse sperm cells. a, Schematic procedure for preparation of total sperm fraction from epididymis. Sperm cells with red or blue heads indicate immature or mature sperm, respectively. **b,** SCSA results of total sperm fractions collected from caudal epididymis. Single sperm cells were identified by To-pro-III (TopIII) staining intensity. **c,** (Left) SCSA results of swim-up sperm from top 0.8 ml medium of 4.0 ml epididymis cultivation. (Right) The mean ± s.d. of percentage of HDS population is shown (n = 3). **d,** The morphology of total sperm and HRCS fractions. **e,** Immunoblot analysis of H3.1 and H3.3 recombinant proteins (1 ng/well) using anti-H3 antibody (ab1791, Abcam) to check the sensitivity of antibody to H3.1 and H3.3. **f,** Immunoblot analysis of H3 in total lysates of mouse embryonic fibroblast (MEF) cells and HRCS fraction.

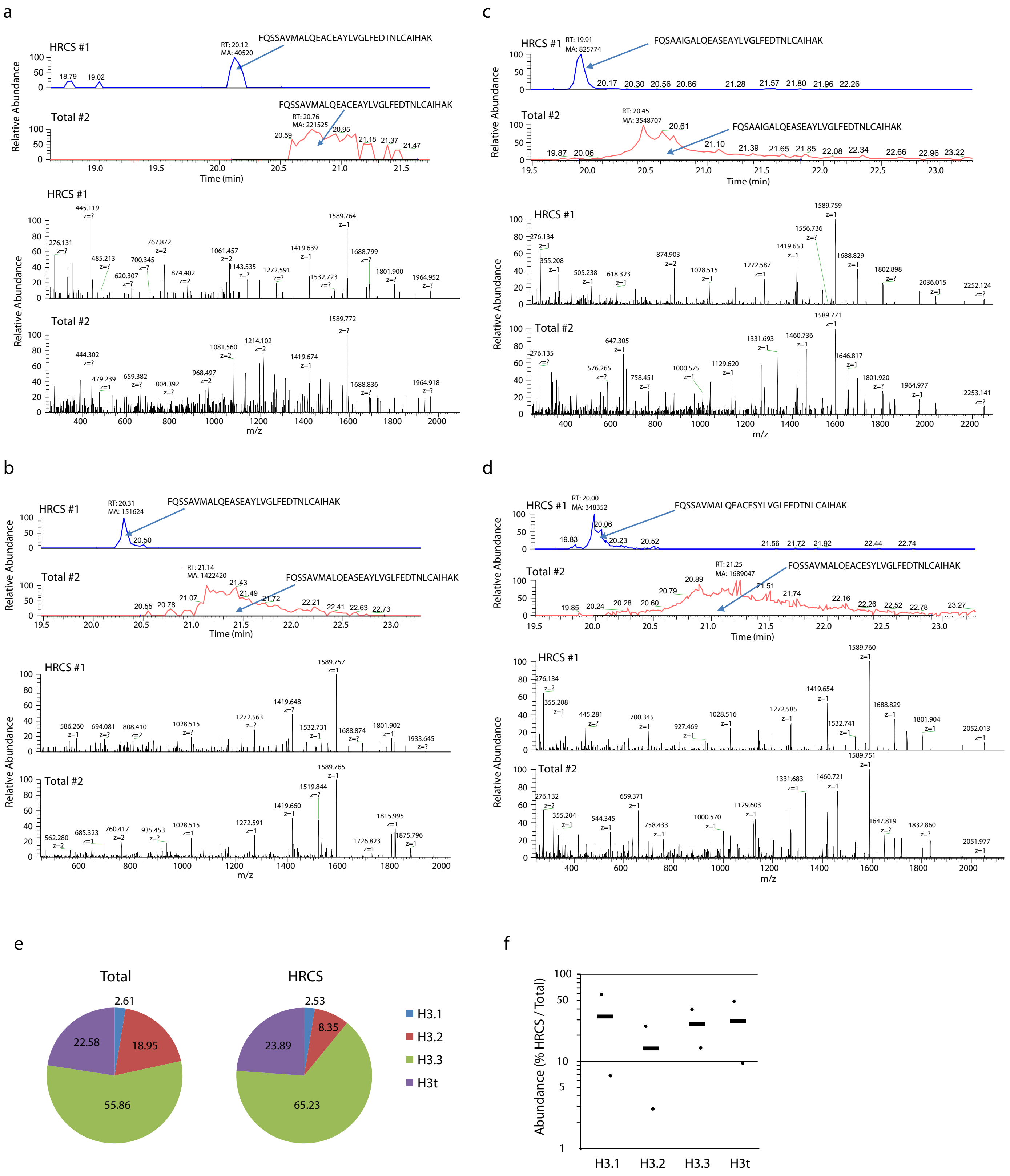

Supplementary Fig. 3 | Measurement of quantitative ratio of histone H3 between total sperm and HRCS fractions by LC-MS assay. a-d, (Top) The MS/MS chromatograms of the y14 ion (m/z=1589.76) of the histone H3 variant specific peptide ions in HRCS and total. (Bottom) The MS/MS spectra of variant specific peptide (a : H3.1, b : H3.2, c : H3.3, d : H3t) from HRCS and total. **e,** The pie chart shows the variety of histone H3 variants in total HRCS. **f,** The dot plot for abundance ratio (%) of HRCS and total in the variants, estimated by the peak areas of MS/MS chromatograms for variant specific peptides.

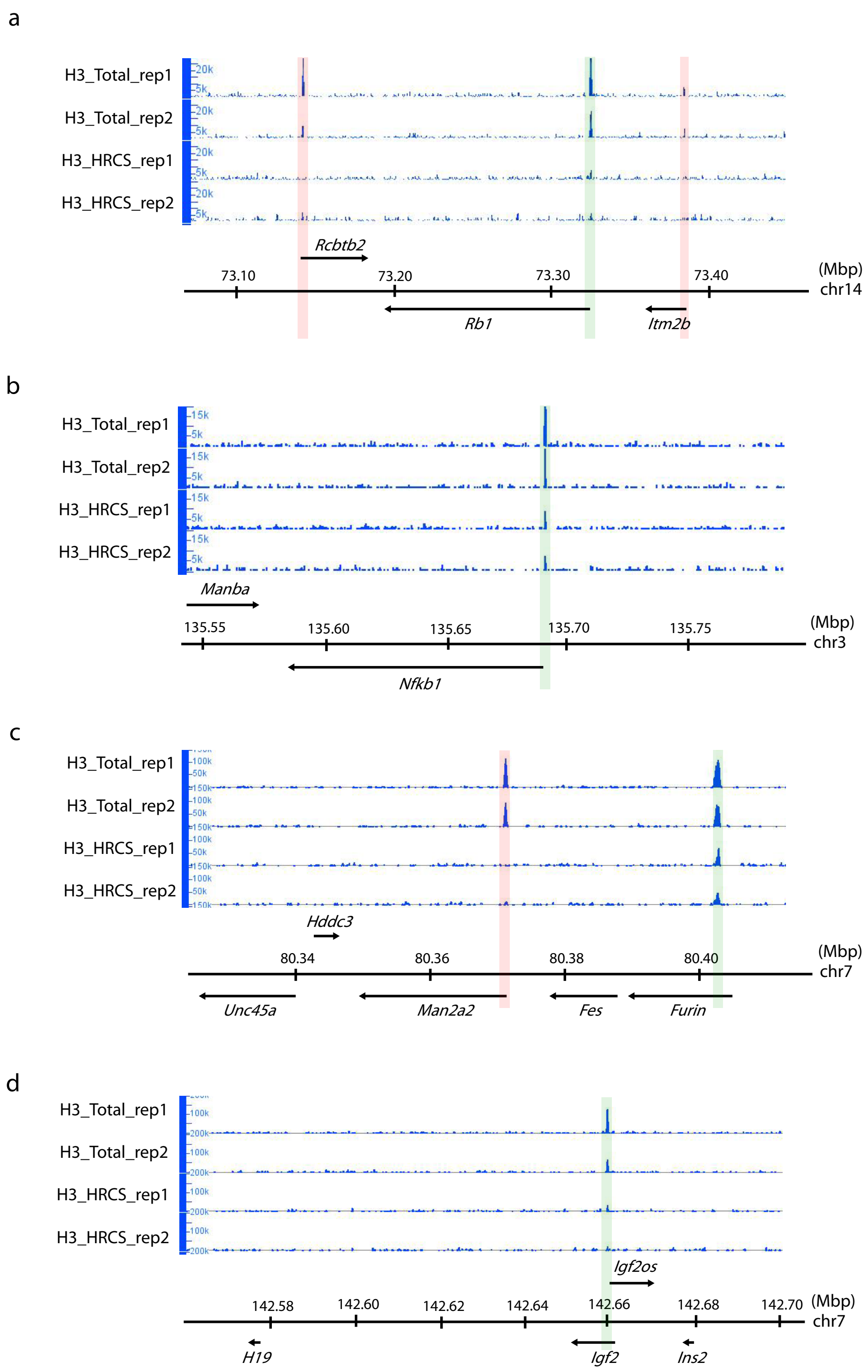

Supplementary Fig. 4 | Raw data of H3 ChIP-seq results using total sperm and HRCS in representative genomic regions. a-d, Blue signal indicates the number of raw reads, which is scaled to mapped regions in each sample. Light blue boxes indicate binding sites detected in both total sperm and CMS. Light red boxes indicate binding sites detected only in total sperm.

a

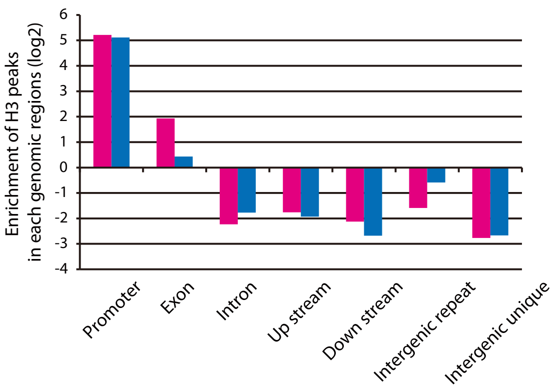

b

H3 peaks in total sperm sample
on intergenic repeat
(896 peaks)

Repeat Name	Num. of peaks
L1Md_T	236
GC_rich	209
C-rich	29
(CCG)n	28
(CGG)n	28
G-rich	24

H3 peaks in HRCS sample
on intergenic repeat
(215 peaks)

Repeat Name	Num. of peaks
L1Md_F2	46
GC_rich	39
(CCG)n	11
L1_Mus3	11
L1Md_T	10
(CGG)n	10

c

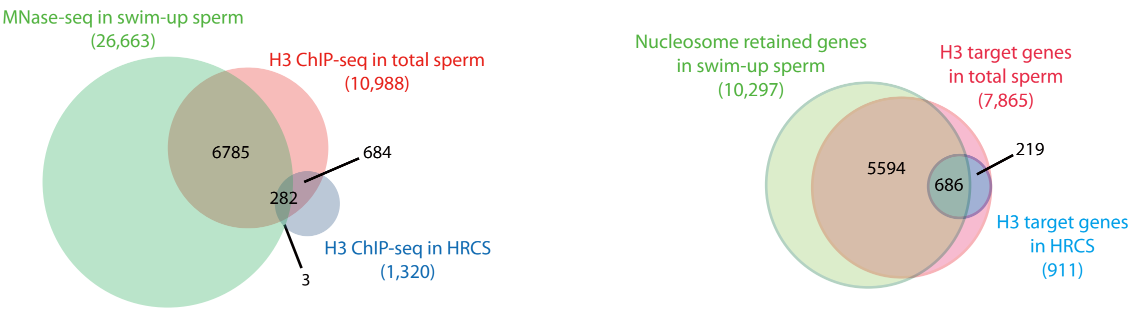

d

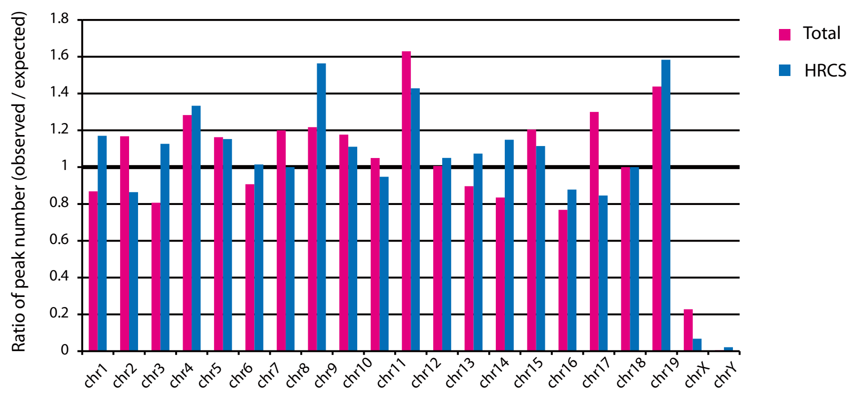

Supplementary Fig. 5 | Analysis for genomic feature of H3-binding site distributions. a, Enrichment of H3 peaks relative to mouse genome background. Enrichment value is presented as log2 scale. **b,** The representative names of repetitive regions included in H3 peaks over intergenic region are shown. **c,** (Left) Comparison of peaks between MNase-seq reported by Erkek et al.[2], and H3 ChIP-seq in total sperm and HRCS. (Right) Comparison of mononucleosome or H3 target genes between MNase-seq in swim-up sperm[2], and H3 ChIP-seq in total sperm and HRCS. **d,** Frequencies of H3 peak number on each chromosome.

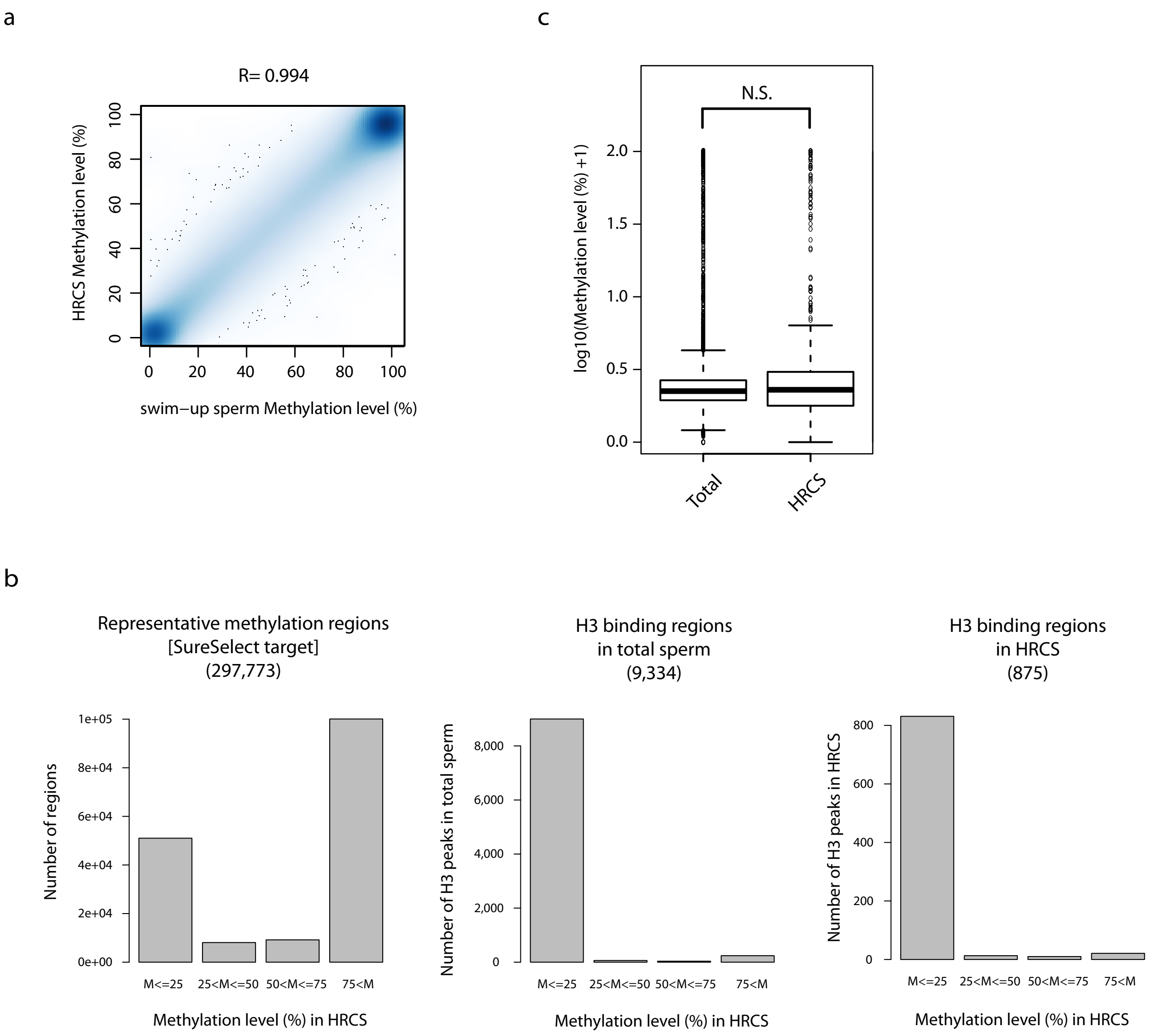

Supplementary Fig. 6 | Correlation of H3-binding sites and DNA methylation status. a, Scatter plot for DNA methylation level between swim-up sperm and HRCS. **b,** Genomic regions targeted by the SureSelect capture probes and H3 peaks in total sperm were divided into four groups by DNA methylation level (%) in HRCS. **c**, Box plot for methylation level (%) in H3 peaks of total sperm and HRCS.

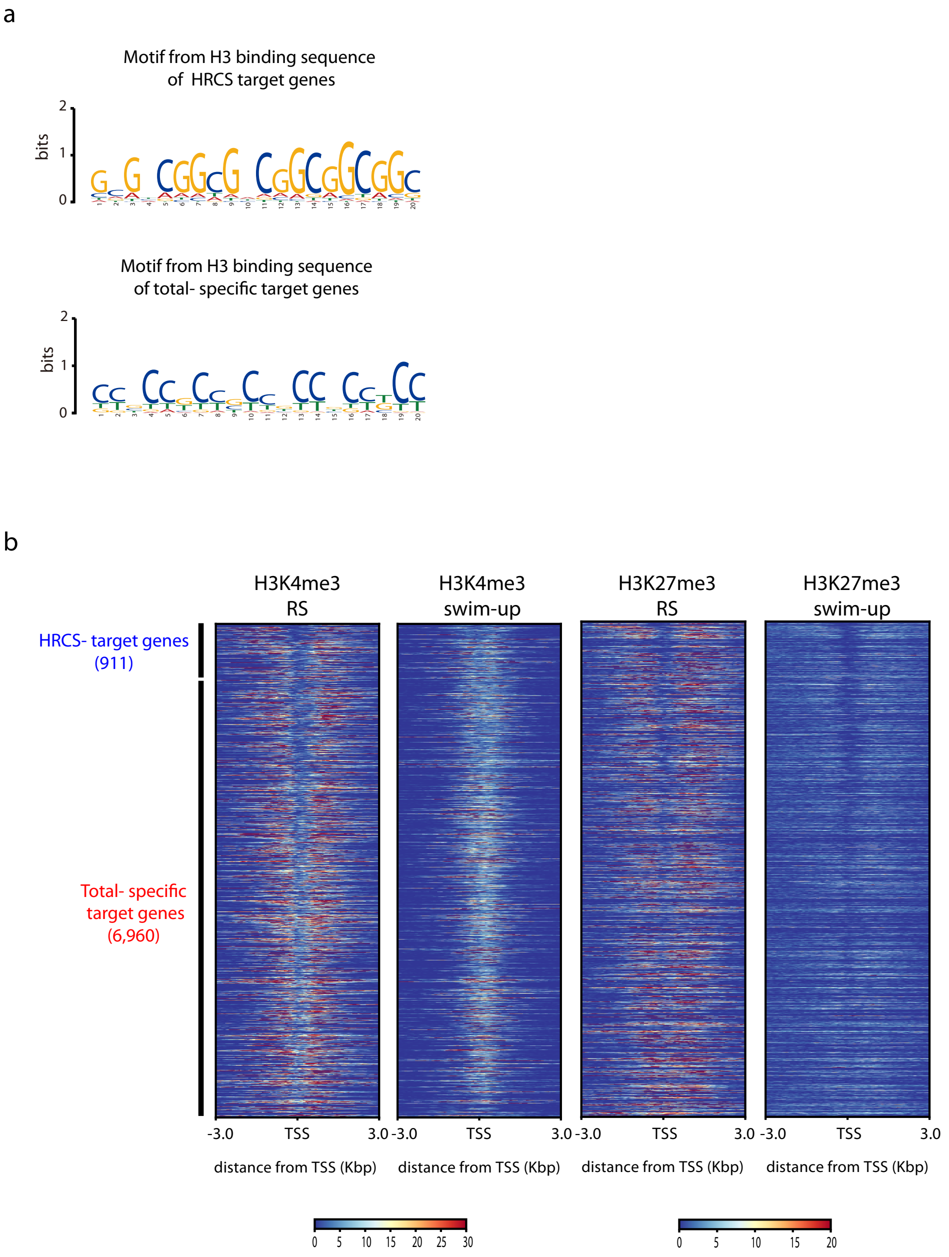

Supplementary Fig. 7 | Binding motif and epigenetic signature for H3 target genes in total sperm and HRCS.

a, The most likely binding motif was extracted from H3-binding sequence of HARCS target genes and total-specific target genes by the MEME algorithm. **b,** Heat map for signal intensity of H3K4me3 ChIP-seq and H3K27me3 ChIP-seq data reported previously[2] in round spermatid and swim-up sperm across H3 target genes in total sperm and HRCS.

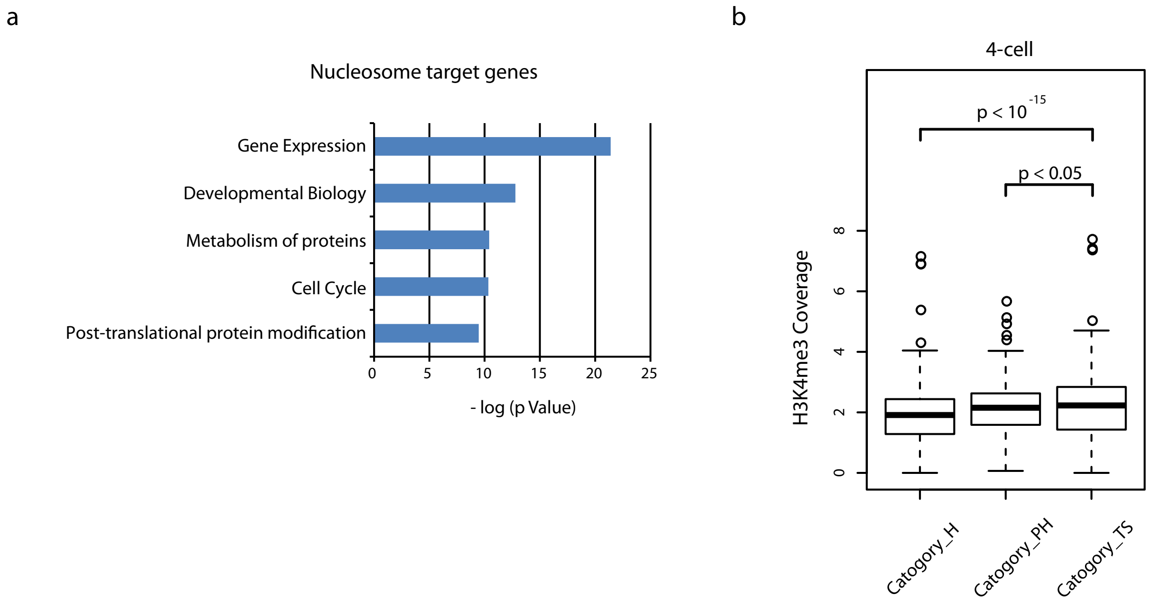

Supplementary Fig. 8 | Relationship between H3 target genes in sperm and epigenetic modification in 4-cell stage

a, Pathway analysis for nucleosome target genes reported by Erkek et al.[2] using Reactome datasets. The five top pathway names are presented. **b,** Box plot for H3K4me3 coverage in downstream region of TSS (between TSS and -0.5kb) for category_H, category_PH or category_TS genes. P values were calculated by Wilcoxon rank sum test.

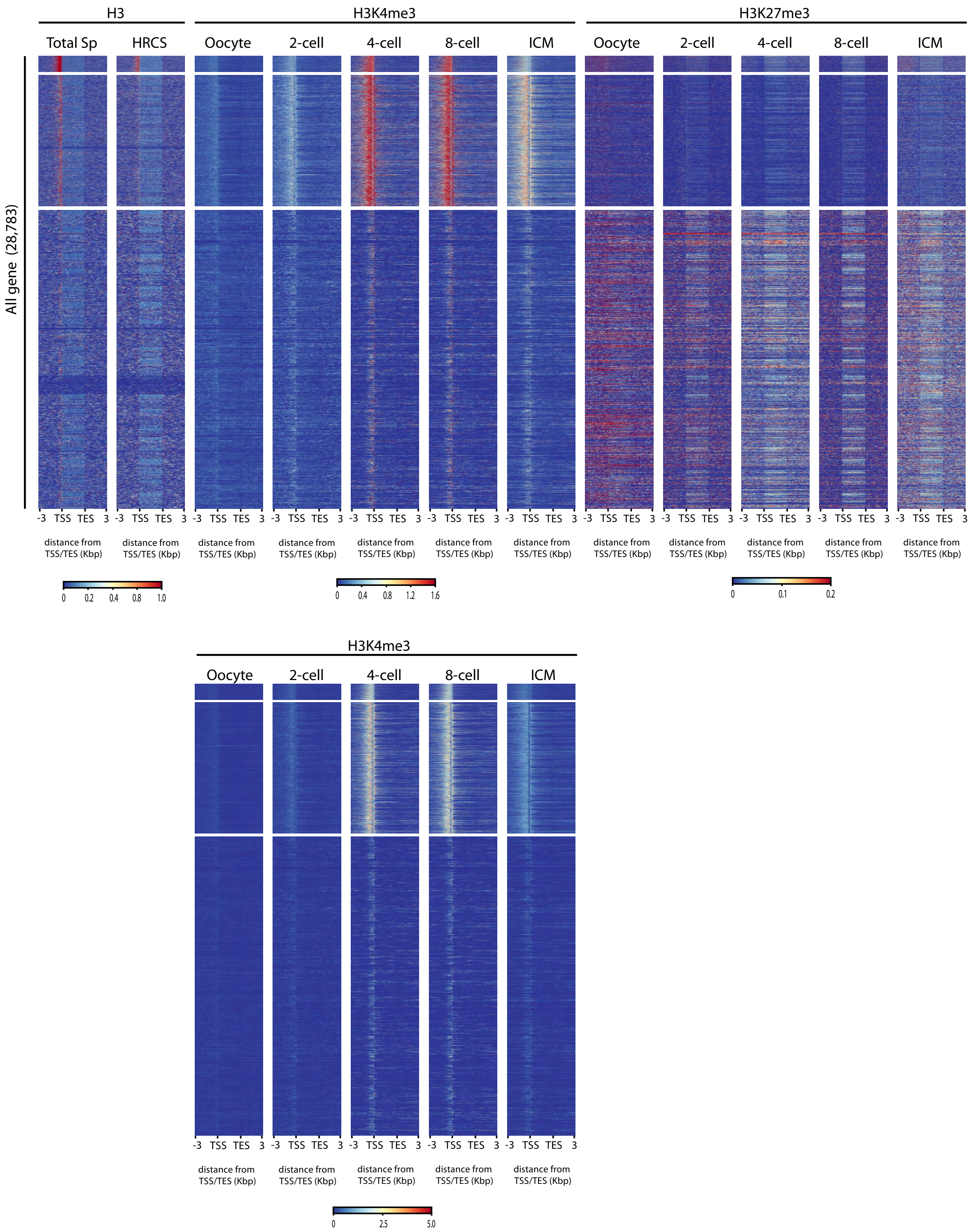

Supplementary Fig. 9 | Dynamics of epigenetic mark in H3 target genes during early embryogenesis. (Top) Heat map for signal intensity of H3K4me3 and H3K27me3 ChIP-seq data[3] from oocyte, 2-cell, 4-cell, 8-cell, and ICM stages across 2 kb around all genes including H3 target genes in total sperm and HRCS, and no H3 target genes in sperm. All genes were categorized into three groups reflecting a specific pattern of each signal by *k*-means clustering. (Bottom) Heat map for signal intensity of H3K4me3 data with different scale is shown to more clearly indicate the difference between genes in HRCS and in total sperm.

a

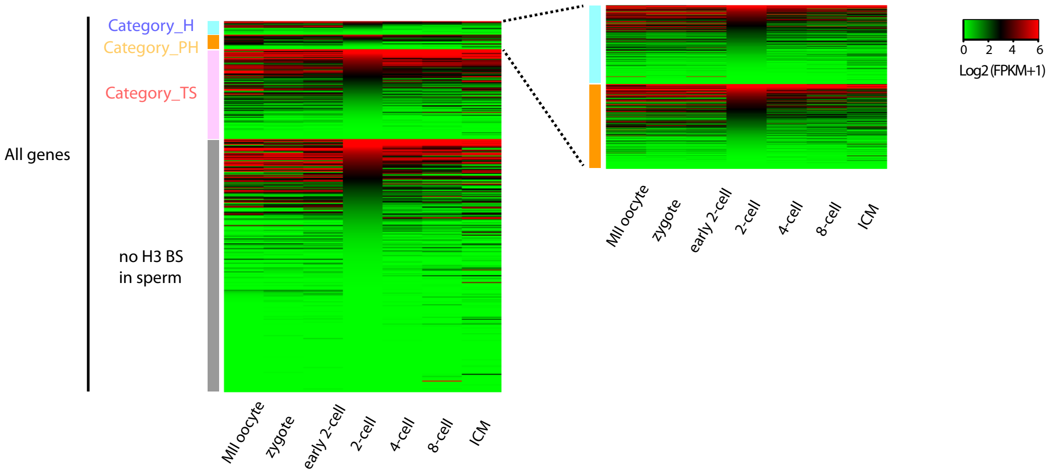

b

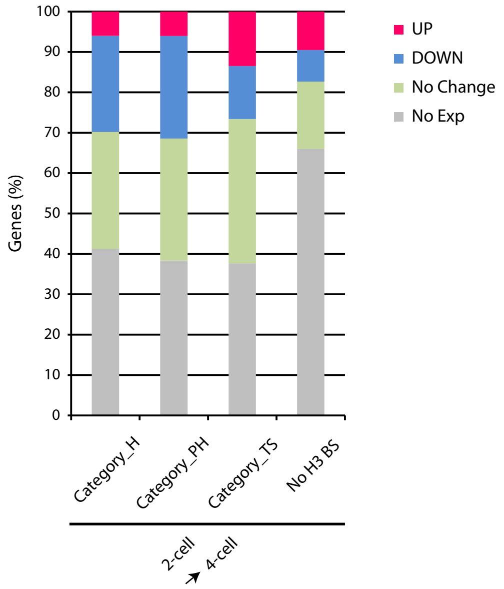

Supplementary Fig. 10 | Dynamics of expression profile in H3 target genes during early embryogenesis.

a, Expression levels of all genes[4] included in category_H/PH/TS are indicated by the heat map. **b,** Population of genes with gene expression changes from the 2-cell to 4-cell stage. The expressional changes were defined as up (fold change > 2), down (fold change < 0.5), no change (0.5 < fold change < 2), and no expression (FPKM < 1).

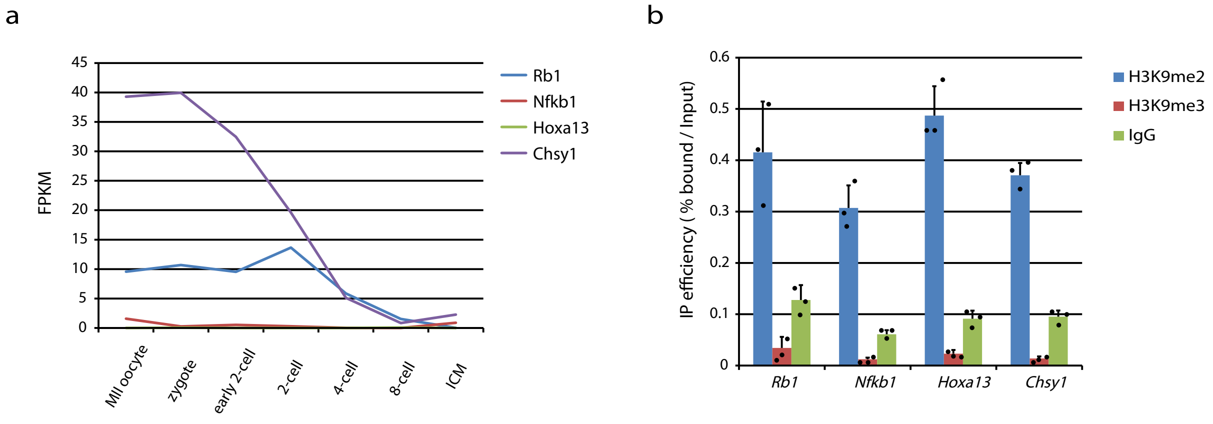

Supplementary Fig. 11 | Analysis for the presence of H3K9me2/3 marks for category_H genes in HRCS.

a, Expression level of 4 category_H genes during early embryogenesis. **b,** ChIP-qPCR result of the abundance of H3K9me2 and H3K9me3 at category_H genes in HRCS. ChIP experiment with non-specific mouse IgG was also performed as negative control. ChIP efficiency was normalized by input DNA sample. Results are from three independent experiments (mean and s.d. of n = 3 mice).

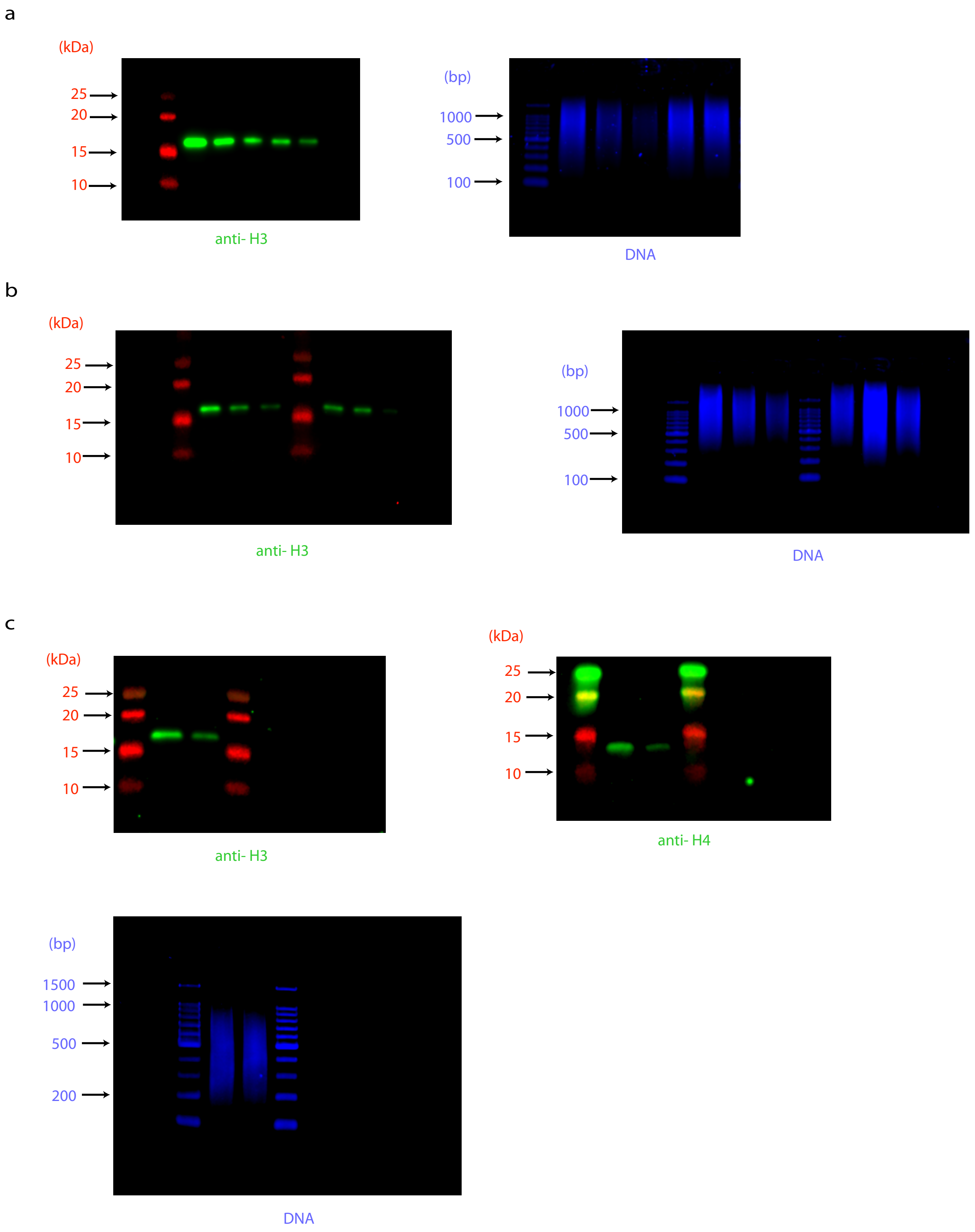

Supplementary Fig. 12 | Uncropped data of western blotting and agarose gel electrophoresis. a, Whole images of Fig. 1b. **b,** Whole images of Fig. 1f. **c,** Whole images of Fig. 2c.

Supplementary Table 1 | List of primers used in this study

Primer Name	Forward	Reverse	Anneal. Temp.
Rb1	ACCCGACTCCCGTTACAAAA	GGCGGAAGTGACGTTTTCC	62°C
Nfkb1	GTGGCGAAACCTCCTCTTCC	CGTCTGTCTGCTCTCTCTCGAC	62°C
Hoxa13	CGTTGTCGTAGAGAAACATGACG	CACTGGGGTCTTCTCCATGC	62°C
Chsy1	ATGCTGCTCGGGCTCGT	GCCCACTCGCTTCAGCTC	62°C

Supplementary Table 2 | Query codes of published datasets used in this study

Experiment	Cell Type	Query Code
H3K4me3 ChIP-seq	Round Spermatid	GSM1046840
H3K4me3 ChIP-seq	Swim-up sperm	GSM1046832
H3K27me3 ChIP-seq	Round Spermatid	GSM1046842
H3K27me3 ChIP-seq	Swim-up sperm	GSM1046834
MNase-seq	Swim-up sperm	GSM1046827
MNase-seq (input)	Swim-up sperm	GSM1046836
ATAC-seq	2-cell	GSM1933924
ATAC-seq	4-cell	GSM1625847
ATAC-seq	8-cell	GSM1933928
ATAC-seq	ICM	GSM1933930
H3K4me3 ChIP-seq	MII Oocyte	GSM2082662
H3K4me3 ChIP-seq	2-cell	GSM2082670
H3K4me3 ChIP-seq	4-cell	GSM2082677
H3K4me3 ChIP-seq	8-cell	GSM2082684
H3K4me3 ChIP-seq	ICM	GSM2082696
H3K27me3 ChIP-seq	MII Oocyte	GSM2082666
H3K27me3 ChIP-seq	2-cell	GSM2082673
H3K27me3 ChIP-seq	4-cell	GSM2082680
H3K27me3 ChIP-seq	8-cell	GSM2082686
H3K27me3 ChIP-seq	ICM	GSM2082698
RNA-seq	Embryogenesis	GSE66582_stage_FPKM

Supplementary References

1. Evenson, D.P. et al. Sperm Chromatin structure assay (SCSA). Methods Mol. Biol. **927,** 147-64 (2013)
2. Erkek, S. *et al*. Molecular determinants of nucleosome retention at CpG-rich sequences in mouse spermatozoa. *Nat. Struct. Mol. Biol.* **20,** 868–875 (2013).
3. Liu, X. *et al*. Distinct features of H3K4me3 and H3K27me3 chromatin domains in pre-implantation embryos. *Nature*. **537**, 558-562 (2016).
4. Wu, J, *et al*. The landscape of accessible chromatin in mammalian preimplantation embryos. *Nature*. **534,** 652-7 (2016).
