# Supplementary material for: Mapping of histone-binding sites in histone replacement-completed spermatozoa
Source: Nat Commun. 2018 Sep 24;9:3885. doi: 10.1038/s41467-018-06243-9 (PMC6155156; doi:10.1038/s41467-018-06243-9)
Supplement: Supplementary file 3 — Description of Additional Supplementary Files [file 41467_2018_6243_MOESM3_ESM.pdf]

Description of Additional Supplementary Files

File Name: Supplementary Data 1
Description: **Results of gene functional analysis for category_H and category_TS target genes**
Pathway analysis for category_H and category_TS genes using Reactome datasets and GO biological process. All the pathway names are presented.
